# A Predictive Model for Cochlear Implant Outcome in Children with Cochlear Nerve Deficiency

**DOI:** 10.1038/s41598-018-37014-7

**Published:** 2019-02-04

**Authors:** Jae Joon Han, Myung-Whan Suh, Moo Kyun Park, Ja-Won Koo, Jun Ho Lee, Seung Ha Oh

**Affiliations:** 1 Department of Otorhinolaryngology-Head and Neck Surgery, Seoul National University Bundang Hospital, Seongnam, Korea; 2 Department of Otorhinolaryngology-Head and Neck Surgery, Seoul National University Hospital, Seoul, Korea

## Abstract

The outcome of cochlear implantation (CI) in patients with cochlear nerve deficiency (CND) is variable, resulting in a wide range of speech perception performance, from degrees of environmental sound perception to conversation without lip-reading. Twenty-five cochlear implantees with CND were enrolled retrospectively to determine the factors correlated with CI outcome in patients with CND and to develop a predictive model for CI outcome. CI outcome was evaluated using the Categories of Auditory Performance (CAP) score at 2 years after CI. Patients with negative auditory brainstem response (ABR) showed a significantly lower CAP score than those with positive ABR (2.5 ± 1.7, 4.8 ± 0.7; p = 0.001). The area ratio of vestibulocochlear nerve (VCN) to facial nerve (FN) at the cerebellopontine angle on magnetic resonance images was positively correlated with CI outcome (p < 0.001). With multiple regression analysis, a predictive equation accounting for 66% of variance of CAP score at 2 years after CI was ${\bf{deduced}}:{\bf{CAP}}\,{\bf{score}}{\boldsymbol{=}}{\bf{0.7}}{\boldsymbol{+}}{\bf{1.9}}{\boldsymbol{\ast }}{\boldsymbol{(}}{\bf{ABR}}{\boldsymbol{)}}{\boldsymbol{+}}{\bf{1.2}}{\boldsymbol{\ast }}(\frac{{\boldsymbol{V}}{\boldsymbol{C}}{\boldsymbol{N}}}{{\boldsymbol{F}}{\boldsymbol{N}}})$. We found that preoperative ABR and area ratio of VCN to FN at the cerebellopontine angle could predict CI outcome in patients with CND. Preoperative counselling based on our predictive model might be helpful to determine treatment modality for auditory rehabilitation and which ear to implant.

## Introduction

Cochlear nerve deficiency (CND) is one of the various causes of hearing loss. It includes cochlear nerve aplasia, absence of cochlear nerve (CN) in internal auditory canal (IAC), and cochlear nerve hypoplasia, which refers to small-sized cochlear nerve in IAC. The size or presence of cochlear nerve could be evaluated using magnetic resonance imaging (MRI). In 1996, Rubinstein *et al*. reported the effectiveness of T2-weighted MRI with oblique parasagittal fast spin-echo^1^. Recently, a more detailed evaluation of the nerves in IAC was made possible using highly contrasted T2-weighted images. In most cases, the size of cochlear nerve was reported to be similar or slightly larger than that of facial nerve (FN)^2^. However, in cases where cochlear nerve is significantly smaller than FN in IAC, it is considered cochlear nerve hypoplasia^3^.

CND is the most common cause of unilateral congenital sensory nerve impairment. It is found in approximately 25–48% of patients with unilateral hearing loss^4–6^. Moreover, bilateral CND is found in approximately 15.4% of patients with severe-to-profound bilateral hearing loss^7^. It has been reported that the hearing threshold and word recognition score of unilateral deafness may be related to the size of bony cochlear nerve canal, which is associated with CND^8,9^. In addition to hearing loss, CND is related to the anomaly of cochlea (27%) and vestibule (49%)^10^.

The outcome of cochlear implants (CI) in patients with CND has been reported to be worse than that of patients with an unknown cause of deafness. Theoretically, the electrical stimulation with CI electrode in the cochlear may not reach the brainstem and auditory cortex in patients with cochlear nerve aplasia^11^. However, CND was not an absolute contraindication to CI due to the limitations surrounding the resolution of MRI and detection threshold in CND, and response of auditory cortex to acoustic stimulation was identified even though cochlear nerve was absent on MR images^12^. The average Categories of Auditory Performance (CAP) score in patients with CND was 4, which indicates discrimination of some speech sounds without lip-reading^13^, and its range was variable from the level of environmental sound detection (CAP 1) to using the telephone with familiar voices (CAP 7)^14–16^. About half of the CI implantees with CND use verbal language as speech alone or augmentation with other communication skills^16,17^, and approximately 10–20% of them use sign language alone without any speech.

Several factors related to the CI outcome in patients with CND have been reported. Speech performance or aided threshold with CI was relatively poor when patients showed cochlear nerve aplasia^14,16,18^, low grades of IAC nerve^16,19^, narrow bony cochlear nerve canal^20^, no response to the intracochlear electrically evoked auditory brain stem response (EABR)^21^, or smaller diameter of vestibulocochlear nerve (VCN) than FN at the cerebellopontine angle (CPA)^21,22^. These factors were helpful for predicting whether the CI outcome would be good. However, a precise prediction for the level of speech performance after CI has not been possible to date.

In this study, we evaluated the speech perception performance of patients with CND after CI and attempted to determine the factors that influence CI outcome. Moreover, we aim to develop a predictive model for CI outcome and treatment strategies in patients with CND.

## Results

### CI outcome

Twenty-five participants (13 males, 12 females) underwent CI at an average of 21.0 ± 10.9 months (range, 12 to 55 months) (Supplementary Table S1). In 18 out of 25 patients (72%), hearing loss was identified with new born hearing screening program at birth. However, in the remaining seven patients (28%), the detection age of hearing loss was variable, ranging from 6 to 43 months.

**Figure 1:**
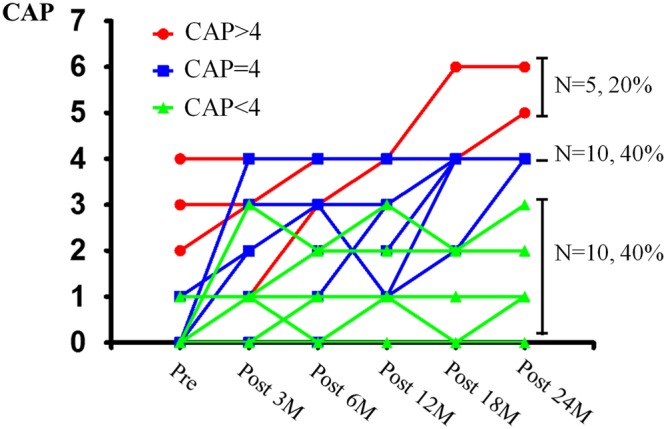
The progression of speech performance after cochlear implant in patients with cochlear nerve deficiency. The Categories of Auditory Performance (CAP) score was variable, ranging from 0 to 6 at 2 years after CI. Among 25 patients, 20% showed good prognosis of CAP 5 or 6, which means the understanding of speech sound without lip-reading (red line). Ten patients showed limited speech perception at the level of CAP 4 discriminating at least two speech sounds (blue line), and the remaining patients showed poor CI outcome and their speech performance was limited to CAP 3 or less, indicating simple recognition of environmental sound or responds to speech sound (green line).

The progression of speech performance was variable after CI (Fig. 1). Among the 25 participants, five patients showed relatively improved speech performance, reaching a CAP score of 5 or 6 (n = 5, 20%), which indicates understanding of common phrases or the ability to carry on a conversation without lip-reading (Fig. 1). Ten patients (40%) showed a CAP score of 4, which indicates the ability to discriminate some speech sounds without lip-reading. The remaining ten patients (n = 10, 40%) showed worse speech performance, with a CAP score of below 3, ranging from 0 to 3, at 2 years after CI. The age at operation among the three groups was not significantly different (27.9 ± 16.0, 16.0 ± 4.2, 21.8 ± 9.3 months, p = 0.23).

### Related factors for CI outcome in CND

The demographic parameters that are known to be related to the outcome of CI, such as age at operation, age of deafness, duration of deafness, and interval to second CI, did not show a significant correlation with speech perception performance, CAP score, and the degree of language development, the Infant-toddler Meaningful Auditory Integration Scale (IT-MAIS), at 2 years after CI in patients with CND (All p > 0.05, Table 1). Eight patients with a positive response to ABR (32%) showed higher CAP score (4.8 ± 0.7) and IT-MAIS (38.5 ± 3.0) than the others with no response to ABR (CAP, 2.5 ± 1.7, p < 0.01; IT-MAIS, 22.3 ± 14.6, p < 0.01). Hearing thresholds, which were evaluated with behavioral audiometry, were not correlated with all the parameters of CI outcome (All p > 0.05) (Table 1). Nine patients underwent promontory EABR preoperatively. The thresholds of EABR ranged from 40 to 500 µA, and were not correlated with a CAP score at 2 years after CI (p = 0.321) (Table 1).

**Table 1:** Bivariate analyses of pre- and peri-operative predictor variables and postoperative speech and language performance.

	CAP score		IT-MAIS	
	R	*P*	R	*P*
Demographic parameters				
Age at operation	0.222	0.287	0.181	0.386
Deaf duration	0.305	0.139	0.105	0.617
Interval to 2nd CI	−0.010	0.969	0.063	0.803
Preoperative audiologic test				
Response of ABR	0.610	0.001*	0.541	0.005*
Hearing thresholds	−0.195	0.350	−0.170	0.417
EABR thresholds	−0.404	0.321	−0.378	0.356
Parameters with imaging				
BCNC size	0.011	0.957	0.066	0.753
IAC size	0.267	0.197	0.319	0.120
VCN size	0.382	0.065	0.455	0.026*
VCN/FN ratio	0.650	<0.001*	0.645	<0.001*
Grade of IAC nerves	0.440	0.028*	0.554	0.004*
Aplasia/hypoplasia	0.257	0.215	0.311	0.131
Postoperative- ECAP				
% of active electrode	0.025	0.905	0.064	0.763
Average thresholds	0.240	0.294	0.096	0.679
Minimum thresholds	−0.160	0.488	−0.255	0.265
Maximum thresholds	0.402	0.071	0.269	0.238
FN twitching with CI stimulation	−0.289	0.160	−0.306	0.136

CAP, the Categories of Auditory Performance; IT-MAIS, The Infant-toddler Meaningful Auditory Integration Scale; CI cochlear implantation; EABR, electrically evoked auditory brain stem response; ABR, auditory brain stem response; BCNC, Bony cochlear nerve canal; IAC, internal auditory canal; VCN, vestibulocochlear nerve; FN, facial nerve; CND, cochlear nerve deficiency; ECAP, Electrically evoked compound action potential; R, Pearson correlation coefficient; *P < 0.05.

With image analysis, the size of bony cochlear nerve canal (BCNC) and IAC was evaluated at the side where the CI device was implanted (Supplementary Table S1). The mean width of BCNC was 0.86 ± 0.53 mm (0–1.87 mm), and most patients (n = 22/25, 88%) had a BCNC that was narrower than 1.4 mm, bordering the normal value of BCNC^23^. The mean diameter of IAC was 3.3 ± 1.2 mm (0.9–5.2 mm). About 68% (n = 17/25) had a diameter of IAC of less than 4 mm, which is the lower limit of normal IAC^24^. The sizes of BCNC and IAC were not correlated with the CAP score (p = 0.96, p = 0.20) and IT-MAIS (p = 0.75, p = 0.12) at 2 years after CI (Table 1). The grade of IAC nerves was correlated with the CI outcome (All p < 0.05) (Table 1). There was a significant correlation between the grade of IAC nerves and the proportion of participants with a CAP score of 4 to 7 (p = 0.04, Linear-by-linear association). Nine patients (n = 9/25, 36%) showed a grade 4 IAC nerves, which indicates cochlear nerve hypoplasia; most of them (n = 8/9, 88.9%) showed a CAP score of 4 or higher. The proportion of patients who showed a CAP score of less than 4 at 2 years after CI was higher in grades 3 (57.1%), 2 (40%), and 1 (75%) than in grade 4 of IAC nerves (11.1%). The presence of aplasia or hypoplasia was not correlated with the CI outcome (p = 0.215) (Table 1). Lastly, we measured the area of FN and VCN at CPA, and the area ratio of VCN to FN was evaluated. The ratio ranged from 0 to 3.3 (1.6 ± 0.8), and it was significantly correlated with the CAP score (p < 0.01) and IT-MAIS (p < 0.01) at 2 years after CI (Table 1).

### A predictive model for CI outcome in CND

In multiple linear regression analysis for CI outcome in patients with CND, preoperative ABR response (p = 0.001) and area ratio of VCN to FN at CPA (p < 0.001) were found to be significant independent factors that affect the CAP scores at 2 years after CI. The R^2^ value for this model was 0.66. Age at operation, duration of deafness, thresholds of EABR, size of BCNC and IAC, area of VCN at CAP, grade of IAC nerves, aplasia/hypoplasia, and results of ECAP were excluded in this model. The predictive equation by step-wise method was:

1 $${\rm{CAP}}\,{\rm{score}}=0.7+1.9\ast ({\rm{ABR}})+1.2\ast (\frac{VCN}{FN})$$

ABR = 1 if presence of ABR response, ABR = 0 if ABR response was not presented

VCA/FN = area ratio of VCN to FN at CPA

**Figure 2:**
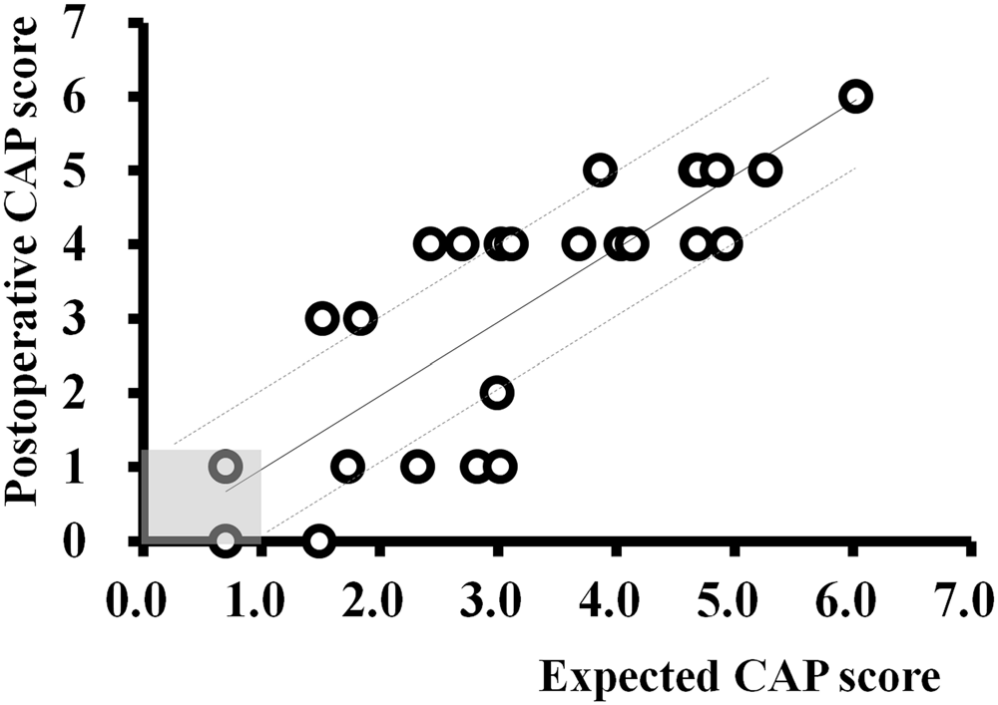
Comparison between expected Categories of Auditory Performance (CAP) score with predictive model and measured speech performance after cochlear implantation (CI). The CAP score, which was expected preoperatively with our predictive model for CI outcome in cochlear nerve deficiency, was highly correlated with the CAP score evaluated at 2 years after CI. The difference between the expected and real CAP scores ranged from 0 to 2.0. When the expected CAP score was less than 1.0 (shaded area), the error between them was limited to be less than 1.0 (area between two dotted lines).

The CAP score at 2 years after CI and predicted CAP score were not significantly different (t, −0.096; p = 0.924) and were positively correlated (Fig. 2; r = 0.812; p < 0.001). The difference between the measured and predicted CAP scores ranged from −2.0 to 1.6 (Fig. 2). The error between expected and postoperatively measured CAP score was limited within ±1.0 when the expected CAP score was less than 1.0. In cases where the expected CAP score was higher than 1.0, the error ranged from −2.0 to 1.6 (0.0 ± 1.1), and its distribution was wider than the others (−0.7–0.3, −0.2 ± 0.7).

## Discussion

We developed a model predicting the postoperative CI outcome in patients with CND using preoperative audiologic results and image findings of MRI. With the response to ABR and the area ratio of VCN to FN at CPA, our model predicted 66% of the variance of postoperative CAP score at 2 years after CI in prelingual deaf children with CND.

The residual hearing level is one of the most important prognostic factors correlated with the outcome of CI^25^. Not only speech perception^26^, but also language development^27^ and communication performance^28^ were better in patients with residual hearing. Residual hearing at low frequencies provides the chance of electro-acoustic stimulation or bimodal hearing, and there were benefits in the perception of speech in difficult sound environments^29–32^ and music perception^33^. A recent study reported that patients with a hearing threshold of less than 70 dB at low frequencies showed better speech perception with bimodal hearing^34^. In our cohort, the average hearing threshold (0.5, 1, 2 kHz) ranged from 78 to 115 dB, and only two patients showed a hearing threshold of 65 dB at 500 Hz. None of our participants tried bimodal hearing or electro-acoustic stimulation due to limited residual hearing and delayed progression of speech perception with CI. Therefore, the correlation between ABR response and CI outcome in patients with CND cannot be explained by residual hearing. Another explanation for the prognostic value of ABR response might be the presence of cochlear nerve. Patients without any ABR response may be associated with having cochlear nerve ‘aplasia’, lower grades of IAC, or smaller area ratio of VCN to FN at CPA. However, ABR response was selected as an independent factor for CAP score at 2 years after CI in multiple regression analysis, and it did not show any correlation with the grade of IAC (p = 0.38, chi-square), aplasia/hypoplasia (p = 0.89, chi-square), and the area ratio of VCN to FN at CPA (p = 0.33, M-W U test).

We speculated that the response of ABR represented the efficacy of hearing aids and cortical development with acoustic stimulation before CI. Residual hearing within 95 dB before CI might result in primitive cortical development by sound stimulation assisted with hearing aids, and it might make the difference of speech perception with electrical stimulation after CI. It has been reported that hearing aids suppressed the negative effect of hearing deprivation on the outcome of CI^35^. The pathologic reorganization of auditory pathway induced by hearing loss might be delayed or inhibited by acoustic stimulation with hearing aids^36^. Therefore, the outcome of CI in patients with CND at 2 years after CI might be related to the ABR response with respect to acoustic stimulation and cortical maturation assisted by hearing aids.

In this study, we hypothesized that the outcome of CI in patients with CND might be related to the size of cochlear nerve. Although the speech performance with CI was known to be unrelated to the remaining number of spiral ganglion cells^37^, patients with CND showed a poorer outcome of CI than those with normal sized cochlear nerve. Furthermore, speech performance was poor in those with cochlear nerve aplasia^14,16,18^ or lower grades of IAC^16,19^ nerves; this means that the amount of cochlear nerve fibers was less than normal sized cochlear nerve. Therefore, the amount of cochlear nerve fiber might be related to the outcome of CI in patients with CND, and the quantitative evaluation of cochlear nerve might be helpful to predict the speech performance after CI.

The quantitative evaluation of the size of cochlear nerve in IAC with MRI was difficult due to the limitation of image resolution and diverse aberrant course of cochlear nerve in CND^38^. In a previous study evaluating the temporal bone pathology, the diameter of cochlear nerve was reported to be correlated with the size of VCN^39^. The cochlear nerve is contained within VCN, which is located at CPA, just outside the midbrain; hence, the size of VCN might indirectly represent the size of cochlear nerve and spiral ganglion neuron^39^. In our study, we measured the area of VCN, instead of the diameter, and evaluated its correlation with the outcome of CI because we speculated that the area of VCN might represent the amount of cochlear nerve fiber more precisely and quantitatively. Considering the variation of subjects and images, we evaluated the area ratio of VCN to FN, which was utilized as the reference for making the diagnosis of cochlear nerve hypoplasia in IAC and CPA^3,14,21,40^.

In our study, the speech outcome of CI in patients with CND showed a significantly positive correlation with the area ratio of VCN to FN, (Table 1) and the parameter was analyzed as an independent factor for the CAP score at 2 years after CI. In this regard, we may be able to predict the postoperative speech performance of CI by evaluating the size of VCN and FN at CPA in patients with CND.

Age at operation and duration of deafness were reported as the most important prognostic factors for CI in congenital deafness^41–47^. The patients who underwent CI before the age of 2 showed better speech perception and language development^42,46,47^, and optimal age for CI has been recommended as 12 months^48,49^. The poor prognosis in patients who underwent CI late was reported to be related with the takeover of auditory cortex resulted by cross-modal brain plasticity^50^, and sufficient auditory stimulation with CI within critical period resulted in restoration of the temporal lobe metabolism by auditory stimuli. On the other hand, even if CI was appropriately performed within critical period, the positive impact of the early implantation on the CI outcome would be eliminated if not enough auditory stimulation was given. Therefore, age at operation and duration of deafness might be excluded from the related factors for CI outcome in patients with CND due to the limited amount of cochlear nerve and auditory stimulation, and the results were consistent with previous studies^15,16,51^.

Preoperative counselling regarding the outcome of CI in patients with CND might be important for both the care-giver and surgeon. Most patients with CND in our study were expected to show poor outcome and low speech perception, with a CAP score of less than 4. Therefore, clinicians should inform the care-giver about the expected outcome of CI and encourage intensive aural rehabilitation with CI. Furthermore, considering the expected CI outcome and progression of speech performance after CI, total communication, lip-reading, or sign language should be considered as alternatives for communication. If the expected speech performance did not reach the level of discrimination of speech sound, aural rehabilitation combined with auditory-verbal therapy and education of sign language might be beneficial to patients with CND. In the future, long-term follow-up results might be necessary to better evaluate the progression of language development with CI and auditory-verbal therapy in patients with CND.

The error between the predicted CAP score and real postoperative CAP score was relatively variable, ranging from −2.0 to 1.6 (Fig. 2). An interesting finding in this study was that the margin of error was wider (−2.0 to 1.6) when the expected CAP score was higher than 1.0, than when the expected CAP score was less than 1.0 (−0.7~0.3). When the speech perception and langue development after CI were nearly absent due to the lack of cochlear nerve, there would be little room to be affected by other factors such as age at operation or duration of deafness. In other hands, the patients with partial deficiency of cochlear nerve showed some progression of speech and langue development after CI, and the factors excluded from our model might have an impact on the CI outcome and hamper the predictability of our model. This variability might be also explained by other factors that were not included in this study, such as family support and socioeconomic status^27,52^. Based on our predictive model, intensive auditory rehabilitation with CI, supported by higher family support, parent-child interaction, and socioeconomic status, might result in better speech performance than the expected outcome. It would be helpful to counsel the parents of children with CND and encourage them to focus more on rehabilitation and support after surgery.

From the surgeon’s perspective, it is important to consider the preoperative prediction of the CI outcome. Having a realistic expectation for CI is necessary to set a good rapport with patients and/or their parents, as well as to decide on an appropriate strategy for aural rehabilitation. Furthermore, if the expected CI outcome is despairing and the progression of speech perception ability seems limited within the expected range, clinicians should consider another treatment option, such as auditory brainstem implant (ABI). In our model, when the expected CAP score was within 1.0, patients showed extremely poor outcome after CI (CAP 0 and 1) (Fig. 2), and the errors between the measured and expected CAP scores were within 1.0 (Fig. 2). To the best of our knowledge, there is no study comparing CI with ABI in patients with CND, and the effectiveness of ABI in non-tumor pediatric patients has not been fully elucidated to date. Nonetheless, the available literature showed that ABI in patients with CND was beneficial and that speech perception without lip-reading was possible^53^. About half of the patients with inner ear anomalies, such as cochlear, labyrinthine, and cochlear nerve aplasia, showed a CAP score of 4 with ABI^54^. In this study, 17% showed relatively good outcome after ABI with a CAP score of 5 or higher, and the other 33% showed a CAP score of 0 to 3. Although the performance with ABI was generally worse than that with CI^55^, some patients with CND may benefit more with ABI than CI^55^. Furthermore, when CI and ABI were sequentially implanted in patients with CND, additive progression of speech perception was reported compared with ABI alone^56^.

**Figure 3:**
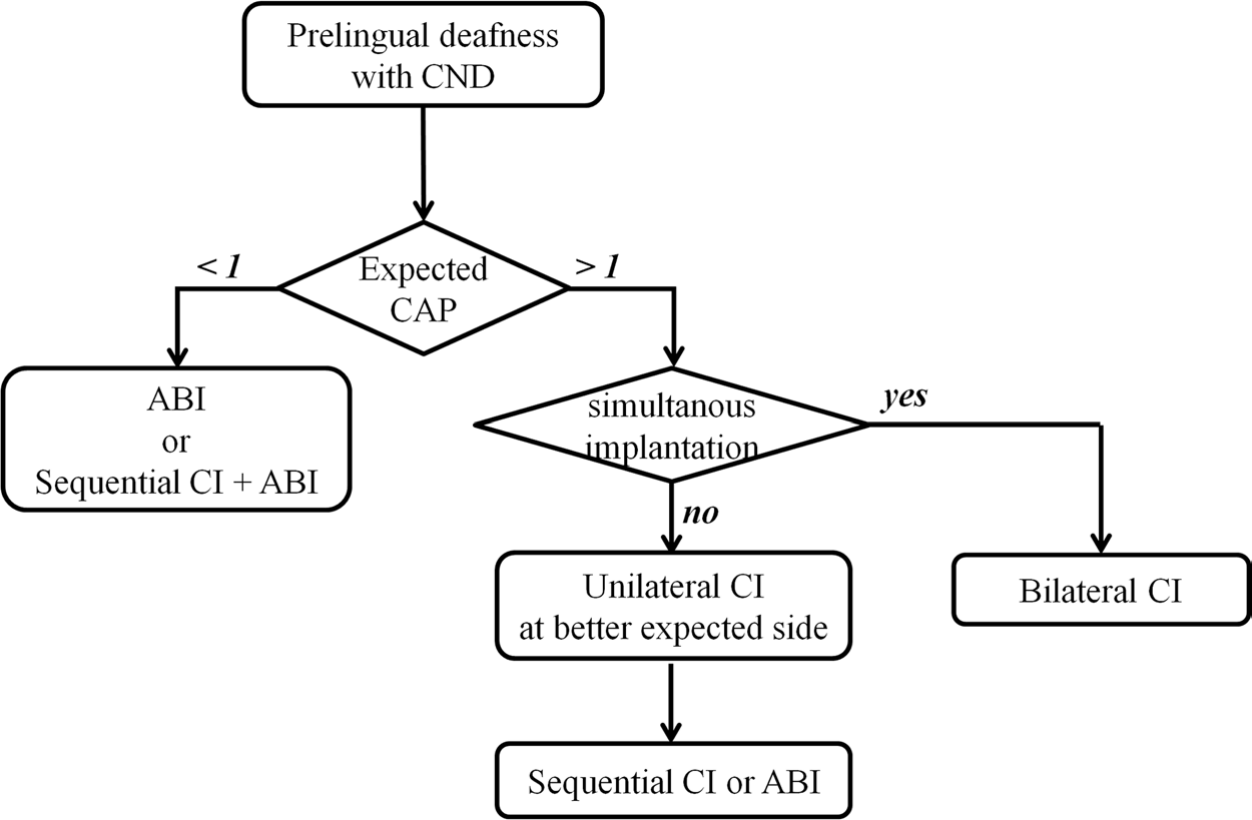
Flow diagram of decision-making process for auditory rehabilitation in patients with cochlear nerve deficiency (CND). This schematic flow diagram represents a protocol for decision-making which treatment modalities and which side to implant in prelingual deafness with CND. When the expected CAP score is less than 1, auditory brainstem implant (ABI) or sequential ABI after cochlear implant (CI) are considered. In other cases (expected CAP >1), simultaneous CI is preferred in patients with CND. If the parents are reluctant to simultaneous CI or if the predicted CAP score is expected to be bad on both sides, unilateral CI at better expected side is considered first. Then, the sequential CI or ABI at contralateral side is considered according to the outcome of first CI and expected CAP score at non-implanted side.

Therefore, our predictive model might be helpful to determine treatment modality for auditory rehabilitation. Unilateral ABI could be considered when the expected CAP score based on equation (1) was less than 1.0 and not superior to the speech performance of ABI in literature (Fig. 3). When the care-giver or parents of the patients were reluctant to ABI due to its invasiveness or incomplete evidence for its effectiveness, or they did not accept the poor CI outcome predicted by our model, sequential ABI at contralateral side after unilateral CI could be considered as another option. In cases where the expected CAP score was higher than 1.0, simultaneous CI is preferred in patients with CND by the reason of effectiveness and less-invasiveness of CI, limited predictability of our model, and importance of binaural hearing (Fig. 2). However, Unilateral CI at better expected side followed by sequential CI or ABI at contralateral side could be considered when poor outcome of CI was expected on both sides preoperatively. According to the outcome of first CI and expected CAP score at non-implanted side, ABI or CI could be considered later at non-implanted side.

Furthermore, the selection of the side for unilateral CI might be determined based on our predictive model. The decision of which ear to implant in unilateral CI has been determined by the residual hearing level, handedness laterality, status of inner ear anomaly, or response of promontory EABR^57,58^. In addition to these factors, our predictive model would provide useful basis for determining the side for unilateral CI in patients with CND, in which the expected CAP was higher than the others (Fig. 3).

We excluded the patients with congenital syndrome, such as CHARGE, severe brain abnormalities, or inner ear anomaly, because those abnormalities of cochlear, brain, and other multiple comorbidities might affect the progression of speech perception performance and language development after CI. Furthermore, the area ratio of VCN/FN could not be utilized in patients with facial nerve abnormality, which was frequently combined with CHARGE syndrome^59,60^. Therefore, clinical and radiological review for the combined other abnormalities should be performed before utilizing our predictive model.

## Conclusion

Speech performance after CI in patients with CND appears to be correlated with audiologic assessments with ABR and with imaging findings of area ratio of VCN to FN at CPA. In our model, the CAP scores at 2 years after CI can be predicted using these factors. Preoperative counselling regarding the outcome of CI in patients with CND based on our predictive model might be helpful to both care-givers and surgeons with respect to developing a realistic expectation for CI and thereby, determining treatment modality for auditory rehabilitation and which ear to implant.

## Methods

### Participants

Twenty-five patients, who underwent CI at Seoul National University Hospital between September 2007 and April 2015, were included. All 25 participants showed severe to profound sensorineural hearing loss on both sides, and bilateral cochlear nerve deficiency was identified preoperatively. Patients with congenital syndrome, such as CHARGE, severe brain abnormalities, or inner ear anomaly, such as common cavity or cochlear aplasia, were excluded. Clinical information regarding age at operation, age of deafness onset, sex, side of operation, duration of deafness, and interval to second CI were evaluated with retrospective review of medical records. Four experienced surgeons – the authors of this article – performed CI. This study was approved by the institutional review board of Seoul National University Hospital (IRB permit no. 1708-024-875). All procedures were performed in accordance with relevant guidelines and regulations. The informed consent was obtained from legal guardians of all participants. All data generated or analyzed during this study are included in this published article and its supplementary information file.

### Audiologic evaluation

The hearing level of participants was evaluated with behavioral audiometry and ABR test. Behavioral hearing thresholds were obtained with warble tones presented at 500, 1000, and 2000 Hz. The hearing level was evaluated with the average of thresholds at these frequencies. ABR test was performed with a click sound; the maximum intensity of the stimulation was 90 dB nHL. The response of ABR test was defined as negative when the V waves were not identified by the highest intensity of stimulation. The promontory EABR was performed preoperatively under general anesthesia with neuromuscular blockade to avoid artifact of FN stimulation^58^. Electrical stimulation of the auditory nerve was performed using a Nucleus Promontory Nerve Stimulator 210012 at stimulation frequencies of 100 Hz according to the previously described techniques^61^. The threshold of EABR was determined based on the presence of Wave V.

All participants underwent intraoperative electrically evoked compound action potential (ECAP) evaluation with electrode located in the cochlear. The thresholds of ECAP response were presented with the current level, which was the minimal electrical stimulation showing positive neural response at each channel. In addition, the ratio of the channels that resulted in positive ECAP response to total number of channels, and maximum/minimum threshold levels among all of the channels were evaluated intraoperatively. Within one month after CI, FN stimulation by current via CI was evaluated with a visible facial muscle spasm, or subjective pain, or tingling sensation on the face while using CI.

### Evaluation of speech perception

The status of speech perception was evaluated preoperatively and postoperatively with the verified Korean version of The Categories of Auditory Performance (CAP) and the Infant-toddler Meaningful Auditory Integration Scale (IT-MAIS) by experienced speech-language pathologist^62,63^. The postoperative speech evaluation was followed at 3, 6, 12, 18, and 24 months after CI.

### Radiologic examinations

The status of cochlear nerve was retrospectively evaluated with images of CT and MR taken before CI. All participants underwent high-resolution temporal bone CT in the axial plane with 0.5–1.0 mm contiguous sections covering the petrous temporal bone. MRI was acquired at 1.5-T or 3.0-T. Highly contrasted T2-weighted axial plane images and oblique sagittal plane scan, perpendicular to the long axis of the IAC, were obtained in all cases.

**Figure 4:**
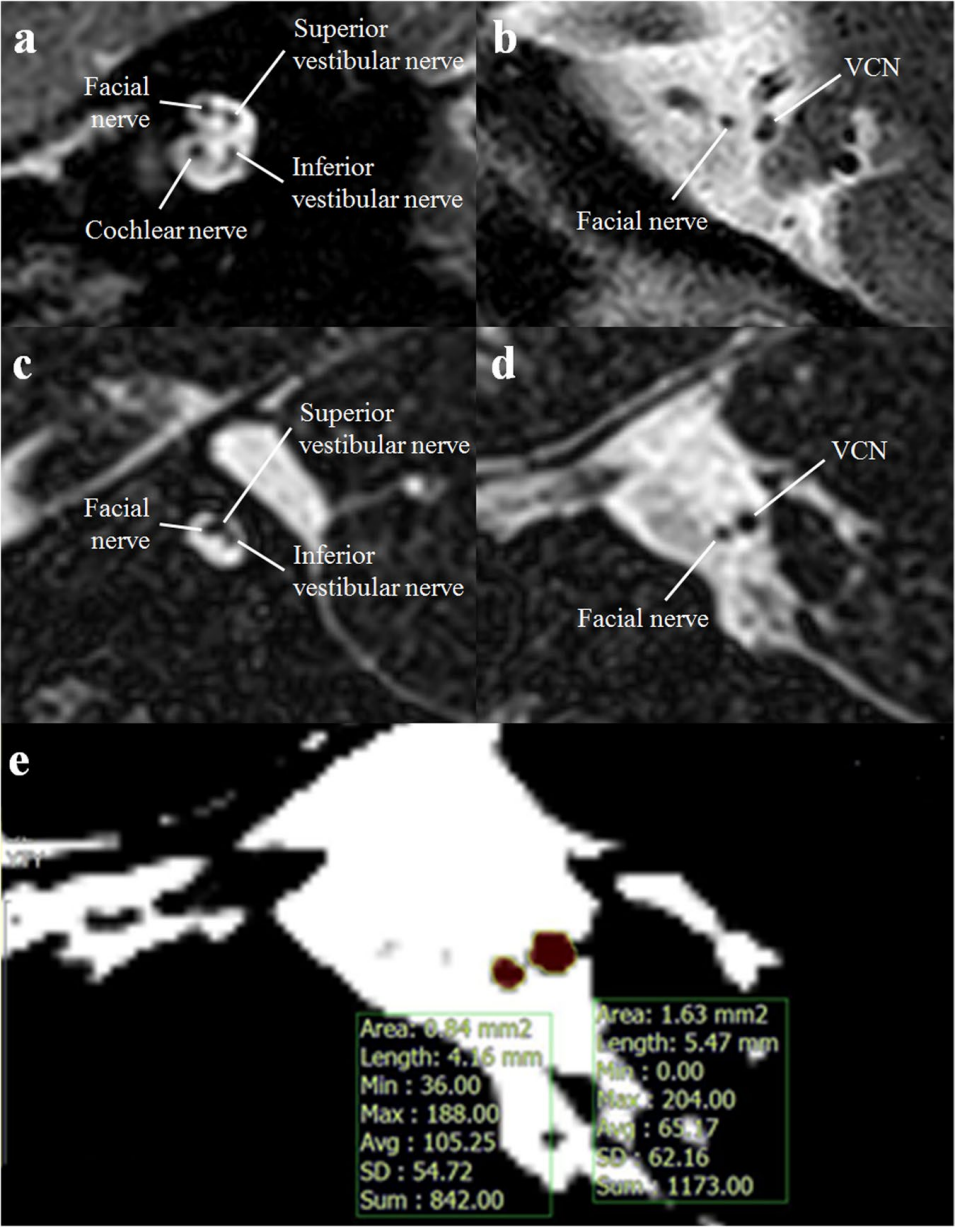
Highly contrasted T2-weighted magnetic resonance images of the internal auditory canal and cerebellopontine angle in patients with cochlear nerve deficiency (CND). (**a**) The image shows four normal sized nerves in the internal auditory canal, indicating cochlear nerve, facial nerve, superior and inferior vestibular nerve at each. (**b**) At the level of cerebellopontine angle, facial nerve and vestibulocochlear nerve (VCN), that emerge from the pontomedullary junction of mid brain, are found. The size of VCN is normal and shows a dumbbell shape. (**c**) The image shows only three filling defect signals in the internal auditory canal, indicating facial nerve, superior and inferior vestibular nerve at each. The cochlear nerve is not found in internal auditory canal, and the patient is diagnosed with CND. (**d**) A small sized VCN with a round shape is identified at the level of cerebellopontine angle in the patients with CND. (**e**) With an image analysis tool, the area of facial nerve and VCN are evaluated (yellow line), and the size of cochlear nerve is indirectly evaluated with the area ratio of VCN to the facial nerve.

All patients were diagnosed and confirmed with MRI as the cochlear nerve aplasia or hypoplasia. Cochlear nerve aplasia was defined as cases in which the cochlear nerve in IAC was not visualized as a bundle definitely in the image review of MRI (Fig. 4c); cochlear nerve hypoplasia was defined as cases in which the attenuation of cochlear nerve was faint or its size was smaller than that of FN in IAC^21^. Another parameter for the evaluation of cochlear nerve was the IAC nerve grading system, which were classified as the number of nerves in IAC: grade 0, no visible nerves in IAC; grade 1, one nerve in IAC; grade 2, two nerves in IAC; grade 3, three nerves in IAC; grade 4, four nerves including hypoplastic cochlear nerve in IAC; grade 5, four normal sized nerves in IAC^16^. The sizes of FN and VCN at CPA were evaluated as the area at the level in which the cross-sections of the nerves were well visualized (Fig. 4d,e). The widest diameter of IAC was measured with the T2-weighted MRI at the implanted site^24^. The size of BCNC was measured with the width of the canal at the midportion of the IAC fundus on CT images^64^.

### Statistical analysis

All results are presented as the means ± standard deviations. Statistical analyses were performed using SPSS (ver. 18.0; SPSS, Chicago, IL). P values < 0.05 were considered to indicate statistical significance. The correlation between possible related factors and outcome of CI was evaluated using Pearson correlation coefficient. Comparisons of the CAP score and IT-MAIS according to the presence of ABR response were performed with the Mann-Whitney U-test. To determine the independent factors predicting the CAP score at 2 years after CI, we performed multiple regression analyses using related factors as independent variables with stepwise selection method.

## Supplementary information

Supplementary table S1.
