# Supplementary material for: A Predictive Model for Cochlear Implant Outcome in Children with Cochlear Nerve Deficiency
Source: Sci Rep. 2019 Feb 4;9:1154. doi: 10.1038/s41598-018-37014-7 (PMC6362156; doi:10.1038/s41598-018-37014-7)
Supplement: Supplementary file 1 — Supplementary table S1. [file 41598_2018_37014_MOESM1_ESM.docx]

**Supplemental information**

**Title: A Predictive Model for Cochlear Implant Outcome in Children With Cochlear Nerve Deficiency**

Jae Joon Han^1^, Myung-Whan Suh^2^, Moo Kyun Park^2^, Ja-Won Koo^1^, Jun Ho Lee^2^, Seung Ha Oh^2,*^

1 Department of Otorhinolaryngology-Head and Neck Surgery, Seoul National University Bundang Hospital, Seongnam, Korea.

2 Department of Otorhinolaryngology-Head and Neck Surgery, Seoul National University Hospital, Seoul, Korea

^*^**corresponding.** **shaoh@snu.ac.kr**

**Supplementary table S1. Clinical and demographic characteristics of the participants.**

|  | Participants (n = 25) |
| --- | --- |
| Demographic parameters |  |
| Sex (M / F) | 13 / 12 |
| Age at operation (months) | 21.0 ± 10.9 |
| Deaf duration (months) | 15.4 ± 7.3 |
| Interval to 2nd CI (months) | 17.0 ± 20.7 |
| Postoperative CI outcome (CAP) |  |
| 12 months | 2.5 ± 1.8 |
| 24 months | 3.2 ± 1.8 |
| Preoperative audiologic test |  |
| Response of ABR  (negative / positive) | 17 / 8 |
| Hearing thresholds (dB) | 98.2 ± 11.1 |
| EABR thresholds (µA) | 228.8 ± 184.0 |
| Parameters with imaging |  |
| BCNC size (mm,) | 0.9 ± 0.5 |
| IAC size (mm,) | 3.3 ± 1.2 |
| VCN size (mm,) | 1.0 ± 0.5 |
| VCN/FN ratio | 1.6 ± 0.8 |
| Grade of IAC nerves  (1 / 2 / 3 / 4) | 4 / 5 / 7 / 9 |
| Aplasia/hypoplasia | 13 / 12 |
| Postoperative- ECAP |  |
| % of active electrode | 39.2 ± 29.9 |
| Average thresholds | 244.9 ± 86.9 |
| Minimum thresholds | 195.6 ± 47.8 |
| Maximum thresholds | 312.3 ± 150.8 |
| FN twitching with CI stimulation  (none / mild / severe) | 14 / 8 / 3 |

CAP, the Categories of Auditory Performance; CI cochlear implantation; EABR, electrically evoked auditory brain stem response; ABR, auditory brain stem response; BCNC, Bony cochlear nerve canal; IAC, internal auditory canal; VCN, vestibulocochlear nerve; FN, facial nerve; CND, cochlear nerve deficiency; ECAP, Electrically evoked compound action potential.
